# Supplementary material for: Topological current divider in a Chern insulator junction
Source: Nat Commun. 2022 Oct 10;13:5967. doi: 10.1038/s41467-022-33645-7 (PMC9550783; doi:10.1038/s41467-022-33645-7)
Supplement: Supplementary file 1 — Supplementary Information [file 41467_2022_33645_MOESM1_ESM.pdf]

Title: Topological Current Divider in a Chern Insulator Junction

Authors: Dmitry Ovchinnikov[1*], Jiaqi Cai[1*], Zhong Lin[1], Zaiyao Fei[1], Zhaoyu Liu[1], Yong-Tao Cui[2], David Cobden[1], Jiun-Haw Chu[1], Cui-Zu Chang[3], Di Xiao[1,4,5], Jiaqiang Yan[6], Xiaodong Xu[1,4§]

Affiliations:
[1]Department of Physics, University of Washington, Seattle, Washington 98195, USA
[2]Department of Physics and Astronomy, University of California, Riverside, California 92521, USA
[3]Department of Physics, The Pennsylvania State University, University Park, Pennsylvania 16802, USA
[4]Department of Materials Science and Engineering, University of Washington, Seattle, Washington 98195, USA.
[5]Pacific Northwest National Laboratory, Richland, Washington, United States
[6]Materials Science and Technology Division, Oak Ridge National Laboratory, Oak Ridge, Tennessee 37831, USA.

*These authors contributed equally to the work.
[§]Correspondence to xuxd@uw.edu

Supplementary Note:

Transport measurement on $MnBi_2Te_4$ junction device

Here we provide additional discussion on the measurement scheme of longitudinal and Hall resistance (R_{xx} and R_{yx}) for each domain. For this measurement, the low-frequency current excitation (V_{ac} ~ 0.05 – 1 V applied to the device in series with 100 MΩ resistor, which corresponds to 0.5 – 10 nA of current) is fed from terminal 1 to terminal 6 and monitored by a virtual earth current preamplifier. The current flows across both domains. In the spirit of four-terminal measurement, contact 2,3,11 (or 4,5,8) that only connects to one domain can be used to read out R_{xx} and R_{yx} as shown in Supplementary Figure 1e.

In the diffusive transport region (V_{bg} < 80 V or V_{bg} > 95 V), the feasibility of R_{xx} and R_{yx} measurement for each domain can be proved by using the theorem of equivalent circuits: when measuring Domain I (or Domain II), the other domain could be treated as the source (or drain) contact resistance. The same equivalence is still applicable when one domain is in a topological transport regime and in the other one transport is diffusive.

When the transport is topological (85 V < V_{bg} < 95 V, C_I = 1 and C_{II} = 2), both domains would have insulating bulk and the majority of transport will be happening on the edge. Now, transport is described by Landauer formula $I_i = \frac{e^2}{h}\sum_j (T_{j,i}V_j - T_{i,j}V_i)$, where $T_{i,j} = N$ when N edge states connect terminal i to terminal j and $V_i(I_i)$ denotes the voltage (current) on terminal i. This leads to a connected current network that ensures the applicability of the above equivalence. To prove this in our case, we have set $I_1 = -I_6 = I_s$ and Landauer formula yields: $V_2 = V_3 = -\frac{1}{2} \times \frac{h}{e^2} I_s$, $V_4 = V_5 = V_6 = -1 \times \frac{h}{e^2} I_s$, $V_7 = V_8 = V_9 = V_{10} = V_{11} = V_1 = 0$, corresponding to our measurement: $R_{xx}^I = \frac{V_4 - V_5}{I_s} = 0$, $R_{xx}^{II} = \frac{V_2 - V_3}{I_s} = 0$ and $R_{yx}^I = \frac{V_8 - V_5}{I_s} = \frac{h}{e^2}$, $R_{xx}^{II} = \frac{V_{11} - V_2}{I_s} = \frac{1}{2} \times \frac{h}{e^2}$.

In Figures 1 e, f, and Supplementary Figure 2 we present raw R_{yx} and R_{xx} data for both domains of Device 1. Due to non-perfect device geometry in exfoliated flakes, unsymmetrized transport data would include mixing between R_{xx} and R_{yx} signals. However, such mixing will not affect the topological transport at the fields above 4 T when the bulk conductivity is negligible.

To perform the directional current measurements as shown in Supplementary Figure 3, the partition of current is dependent on the load of each chiral edge state (CES). We note that the difference between the load of two CES (load 8 and load 9 in this case) is negligible. The total resistance of the load is ~800Ω << h/e^2.

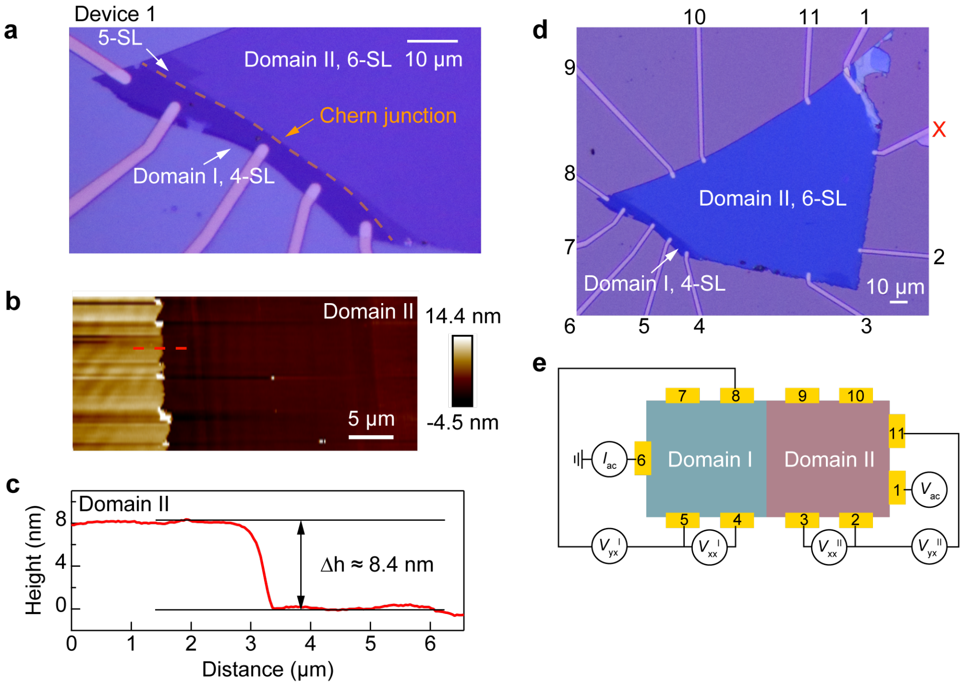

Supplementary Figure 1 | Thickness determination for Device 1. a, Optical micrograph of Device 1. Domain I and Domain II are denoted. **b,** Atomic force microscopy micrograph of the edge of Domain II. **c,** Atomic force microscopy linecut of the edge of Domain II with estimated thickness of ~8.4 nm, corresponding to 6 layer device with ~1.4 nm per layer thickness. **d,** Optical micrograph of Device 1 with contacts indicated. **e,** Schematic of transport measurements for Device 1. Constant AC current is applied from terminal 1 and detected at terminal 6. Longitudinal (V_{xx}^{I}, V_{xx}^{II}) and Hall (V_{yx}^{I} and V_{yx}^{II}) voltages are measured between corresponding electrodes.

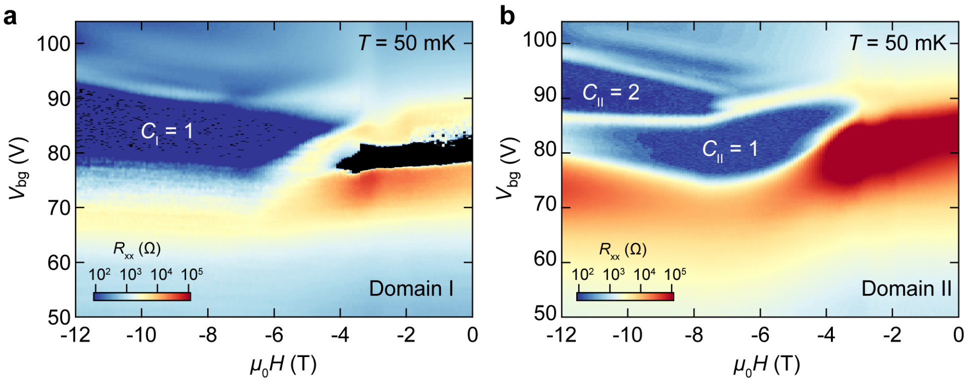

Supplementary Figure 2 | 2D color maps of raw longitudinal resistance R_{xx} as a function of magnetic field $\mu_o H$ and back gate voltage V_{bg} for Device 1. a, Domain I and **b,** Domain II. $T = 50$ mK. In the region 80 V < V_{bg} < 85 V and -4.2 T < $\mu_o H$ < 0 T, Domain I becomes too resistive for lock-in type measurements (area marked with black color).

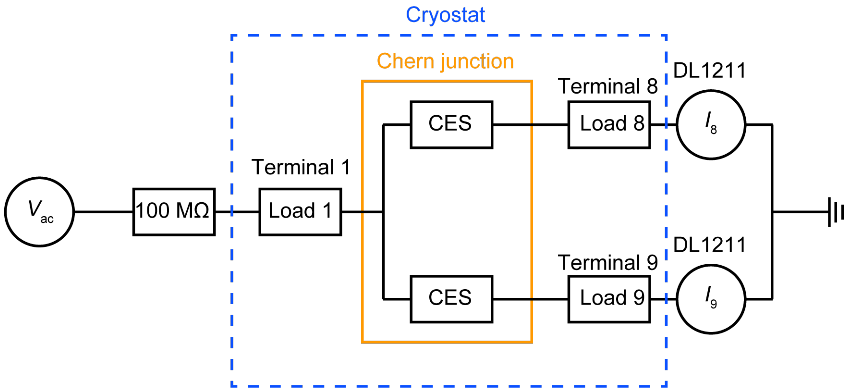

Supplementary Figure 3 | Schematic of measurement. This measurement schematic corresponds to Figure 2a, b of the Main Text. Low frequency (f between 7 Hz and 20 Hz) AC voltage V_{ac} (0.2 V – 0.4 V) is applied from SR830 lock-in amplifier and passed through 100 MΩ resistor for constant current measurements. Current is further applied to terminal 1 (R_{load1} = R_{line} + $R_{filters}$ + R_{C1}, where R_{C1} is contact resistance of terminal 1, R_{line} is resistance of wiring inside the cryostat, R_{filter} is resistance of low temperature filters) and is split into two identical current paths each going through chiral edge state. Next, current is detected through terminal 8 and terminal 9 with two virtual-earth current preamplifiers. Load 8 and Load 9 are defined in the same way as Load 1.

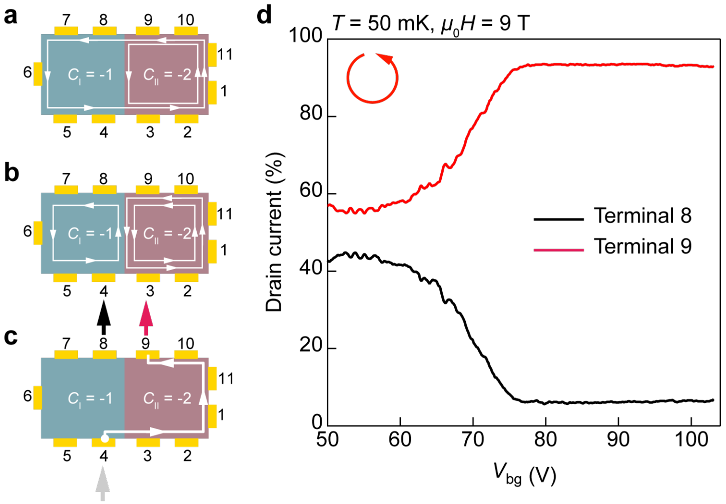

Supplementary Figure 4 | a, Schematic of edge states at positive magnetic field in the regime of C_I = -1 and C_{II} = -2. **b,** A different physical picture, where C_I = -1 and C_{II} = -2 are decoupled. Single edge state exists in Domain 1 with the direction pointing up at the boundary. Two edge states exist in Domain II with direction pointing down. **c,** Schematic of directional current measurement. Current is injected from terminal 4 and detected from terminals 8 and 9. **d,** Normalized drain current at terminals 8 and 9, corresponding to the schematic in **c**. T = 50 mK, $\mu_0 H$ = +9 T. Top inset shows the winding direction of edge state. This measurement also confirms the physical picture depicted in panel **a**. If physical picture depicted in panel **b** would take place, terminals 4 and 8 should be directly connected through the edge state in Domain I, opposite to the observation.

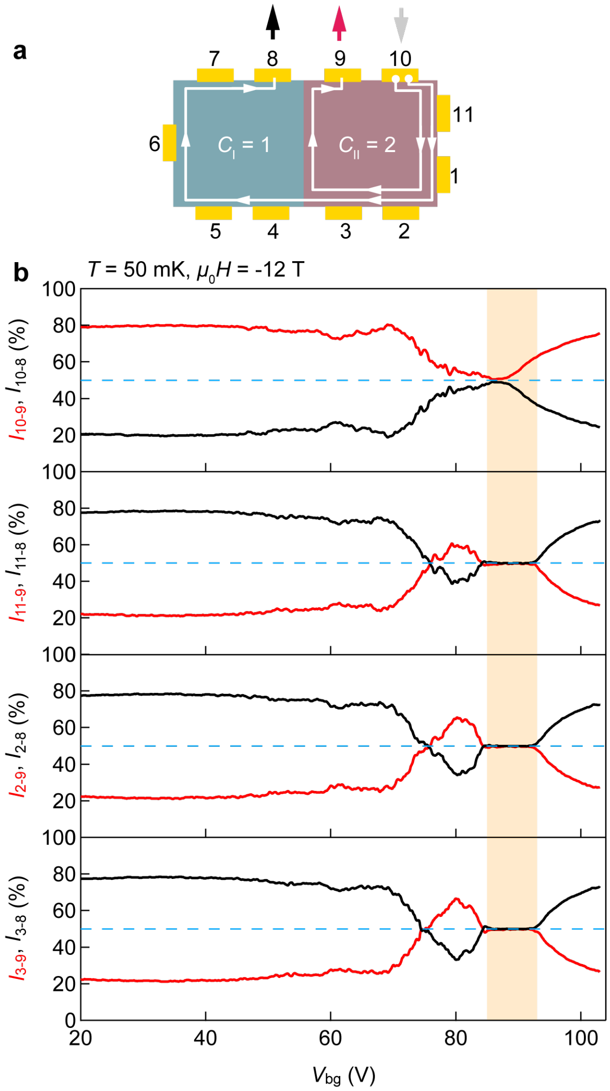

Supplementary Figure 5 | Measurement of Device 1 with different pairs of electrodes. a, Example of current path, where current is injected at terminal 10 and detected at terminals 8 and 9. **b,** Current measured at various configurations, where current injection terminal in Domain II is varied between 10, 11, 2, and 3, while detection terminals are fixed at 8 (Domain I) and 9 (Domain II). $\mu_0 H$ = -12 T, T = 50 mK. Winding direction is clockwise.

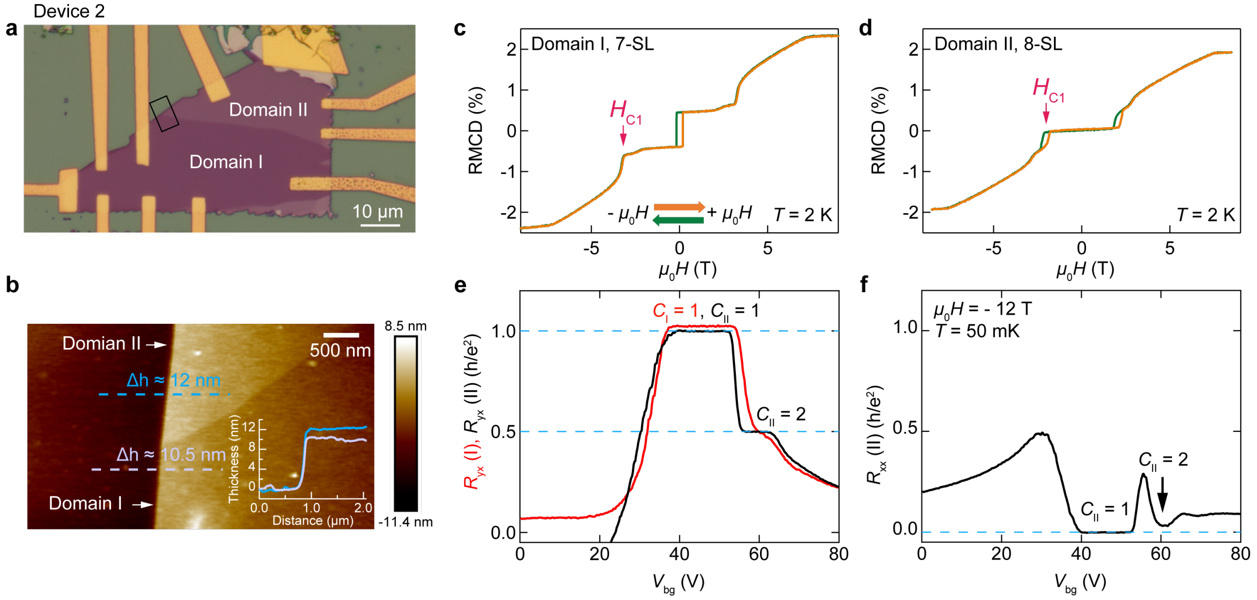

Supplementary Figure 6 | Chern insulator junction Device 2. a, Optical micrograph of the device. **b,** Atomic force microscope image of the area denoted by black rectangle in **a**. Inset shows linecuts along the edge of Domain I and Domain II. **c-d,** RMCD sweeps as a function of magnetic field for **c** - Domain I and **d -** Domain II. H_{C1} denotes the critical field for spin flop transition. Green arrow shows magnetic field sweep down, orange arrow – magnetic field sweep up. **e,** R_{yx} versus V_{bg} at fixed μ_0H = -12 T. Red and black traces correspond to Domain I and Domain II, respectively. **f,** R_{xx} versus V_{bg} at fixed μ_0H = -12 T for Domain II.

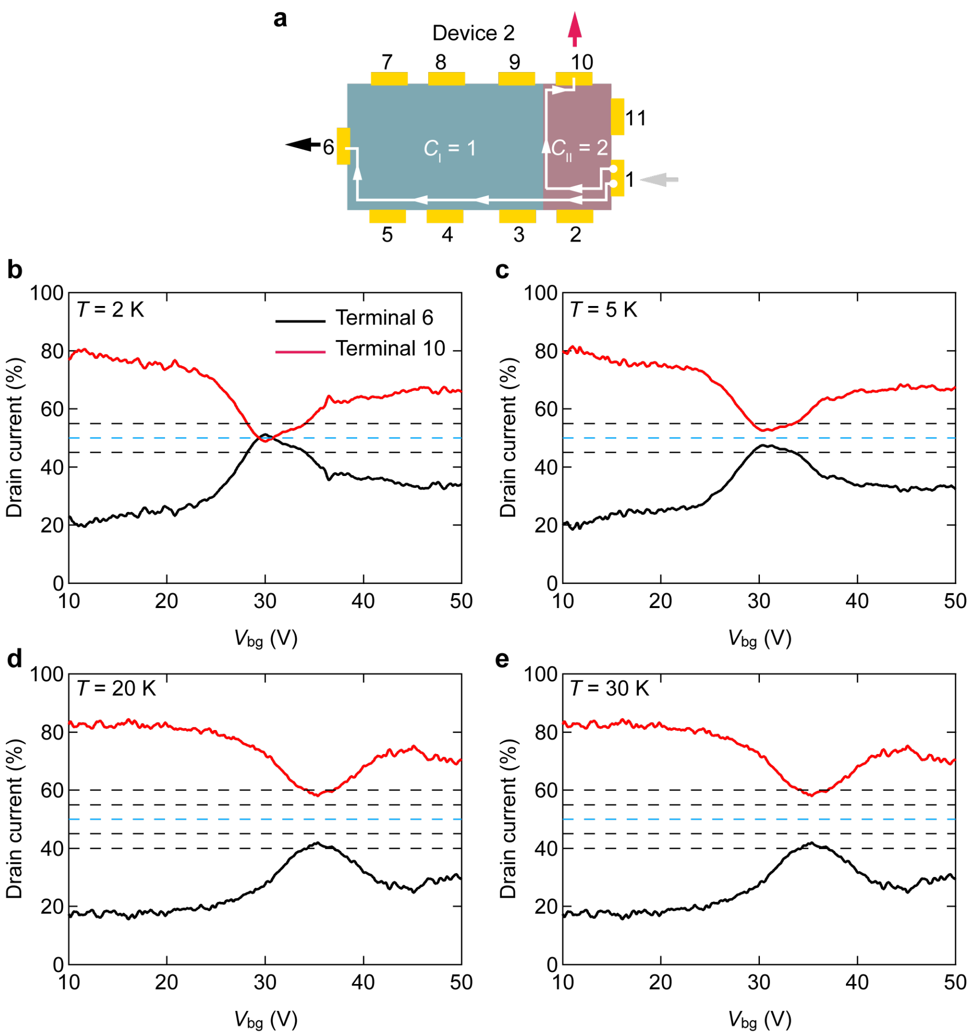

Supplementary Figure 7 | Temperature dependent measurement of Device 2. a, Measurement schematic. **b-e,** Temperature dependence of current divider performance. $\mu_0 H$ = -9T.
